# The Association between Chronotype and Dietary Pattern among Adults: A Scoping Review

**DOI:** 10.3390/ijerph17010068

**Published:** 2019-12-20

**Authors:** Fatin Hanani Mazri, Zahara Abdul Manaf, Suzana Shahar, Arimi Fitri Mat Ludin

**Affiliations:** 1 Dietetic Program and Centre for Healthy Aging & Wellness, Faculty of Health Sciences, University Kebangsaan Malaysia, Jalan Raja Muda Abdul Aziz, Kuala Lumpur 50300, Malaysia; fatinhananimazri@gmail.com (F.H.M.); suzana.shahar@ukm.edu.my (S.S.); 2 Biomedical Science Program and Centre for Healthy Aging & Wellness, Faculty of Health Sciences, Universiti Kebangsaan Malaysia, Jalan Raja Muda Abdul Aziz, Kuala Lumpur 50300, Malaysia; arimifitri@ukm.edu.my

**Keywords:** chronotype, circadian preference, dietary behaviour, nutrient intake, food group intake

## Abstract

Chronotype reflects an individual’s preferred time of the day for an activity/rest cycle and individuals can be classified as a morning, intermediate, or evening type. A growing number of studies have examined the relationship between chronotype and general health. This review aimed to map current evidence of the association between chronotype and dietary intake among the adult population. A systematic search was conducted across five databases: EBSCO Host, Medline & Ovid, Pubmed, Scopus, and The Cochrane Library. The inclusion criteria were adult subjects (more than 18 years old), and included an assessment of (i) chronotype, (ii) dietary behaviour/nutrient intake/food group intake, and (iii) an analysis of the association between chronotype and dietary behaviour/nutrient intake/food group intake. A total of 36 studies were included in the review. This review incorporated studies from various study designs, however, the majority of these studies were based on a cross-sectional design (*n* = 29). Dietary outcomes were categorized into three main groups, namely dietary behaviour, nutrient intake, and specific food group intake. This scoping review demonstrates that evening-type individuals are mostly engaged with unhealthy dietary habits related to obesity and were thus hampered in the case of weight loss interventions. Hence, this review has identified several dietary aspects that can be addressed in the development of a personalised chrono-nutrition weight loss intervention.

## 1. Introduction

An organism develops its own timekeeping system to anticipate and adapt to the day–night cycle of the earth’s 24 h day, known as circadian rhythm [1]. It is generated by the central circadian clock located in the suprachiasmatic nucleus (SCN) of the anterior hypothalamus in the brain [2]. This internal body clock system comprises approximately 24 h, ranging from 23.27 to 24.64 h among individuals [3]. Hence, the daily alignment between the circadian clock and day–night cycle is very important, with light being the most potent external time signal or zeitgeber [4,5,6]. There are various circadian phase markers, and one of these is known as chronotype or circadian preference, which reflects an individual’s preferred time of the day for an activity/rest cycle. Individuals can be categorised as being a morning, intermediate, or evening-type [7,8,9].

Chronotype has often been linked to dietary intake, especially among the evening type. Individuals with a late chronotype exhibit lower adherence towards a healthy diet [10], delay in meal timing [11], a habit of skipping breakfast [12], lower consumption of fruit and vegetables [8], and a higher preference for sugary food/beverages and alcohol [13]. It is well-known that diet quality affects an individual’s health status [14,15]. Indeed, a higher calorie intake during dinner and a shift in meal time towards a later time of the day have been associated with increased odds of developing obesity [16,17]. Conversely, frequent breakfast skipping was shown to affect postprandial insulin concentration, and possibly jeopardise glucose homeostasis in the long term [18]. Many studies have therefore been conducted to investigate the association between diet and chronotype health [12,19,20,21].

Despite the increase in the number of studies investigating this aspect of chronotype, to date, there remains a paucity for a complete and comprehensive review mapping the evidence of this association. Hence, the aim of the present scoping review was to investigate the association between chronotype, dietary behaviour, and overall diet quality among adults. Furthermore, this review also aimed to systematically collate a database of relevant articles covering this area and to become a basis for future systematic reviews.

## 2. Materials and Methods

This scoping review followed the methodology outlined by Arksey, & O’Malley (2005) [22]. The primary review question—what is the association between chronotype and dietary intake?— was the basis for this scoping review. The search reporting was adapted from the preferred reporting items for systematic reviews and meta-Analyses (PRISMA 2009) checklist.

### 2.1. Search Strategy

A literature search was conducted in March 2019. A preliminary scoping search was conducted to determine the feasibility of this review. Once the search strategy was developed, it was independently verified by two reviewers. The following databases, EBSCO Host, Medline & Ovid, Pubmed, Scopus, and The Cochrane Library, were systematically searched to identify relevant publications. All the identified studies were screened for eligibility based on the information contained in the title and abstract. No restriction was set on the year of publication; however, studies were limited to those published in the English language and conducted among human subjects only.

The search terms used for this review were:

1. Chronotype OR circadian preference OR morningness OR eveningness OR circadian timing OR chronobiological
2. Dietary behaviour OR eating habit OR dietary intake OR food intake OR meal consumption OR diet quality OR macronutrient OR micronutrient OR meal timing OR food preference OR portion size OR appetite OR craving OR night eating syndrome OR binge eating
3. 1 AND 2

### 2.2. Selection Criteria

All studies that reported an association between chronotype and dietary aspects, irrespective of study design (except for letters to the editor and conference proceedings with abstract only), were eligible for this review. However, this scoping review only included studies that were conducted among adults (more than 18 years old) and assessed the circadian rhythm phase as a chronotype. Studies conducted among animals or among individuals with a sleep disorder, mental illness, depression, bipolar disorder, and blind individuals were all excluded.

### 2.3. Selection of Included Publications

**Figure 1:**
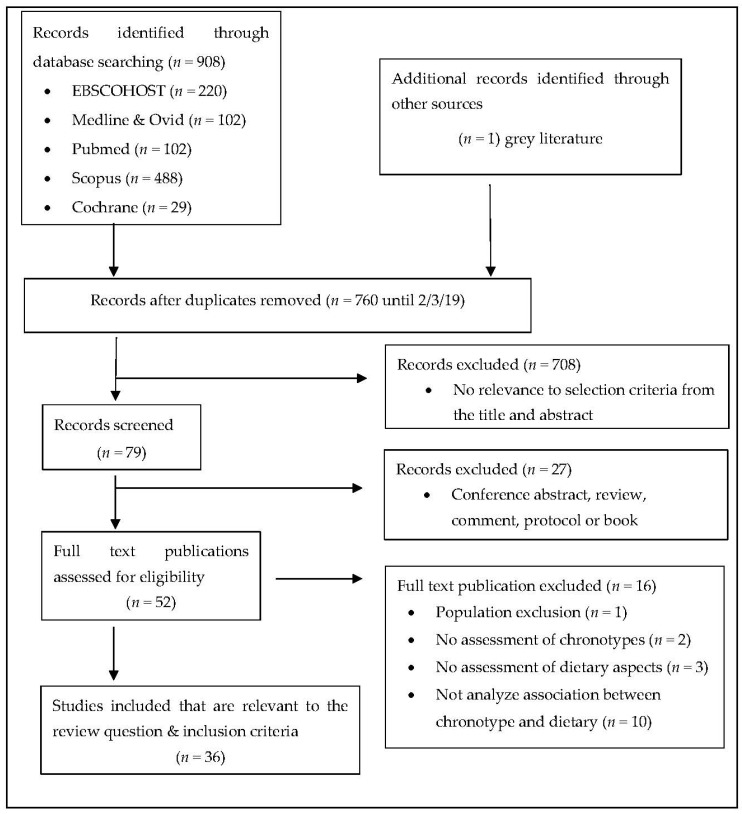
PRISMA 2009 flow diagram.

All the studies that were identified through database search were screened for eligibility by scrutinising the title and abstract of each listed study. The full text of potential eligible studies was retrieved for further screening of their suitability based on the review question and inclusion criteria. The process of selection is outlined in Figure 1. Unlike other scoping reviews, experts were not consulted for this current review.

## 3. Results

A total of 36 articles reporting on 35 studies were included in this review (Table 1). The included studies varied from 12 countries with the majority of the study conducted in Japan (*n* = 7), followed by the USA (*n* = 5), Brazil (*n* = 5), Spain (*n* = 5), Finland (*n* = 4), German (*n* = 2), Turkey (*n* = 3) Korea (*n* = 1), Malaysia (*n* = 1), Thailand (*n* = 1), China (*n* = 1), and India (*n* = 1). Most of the studies were of a cross-sectional study design (*n* = 29), whilst the remaining studies were either interventions (*n* = 2), prospective studies (*n* = 2), prospective randomised controlled studies (*n* = 1), or follow-up studies (*n* = 1). The total number of participants reviewed were 477,493 (Range: 66–439,933 participants) with six studies that were exclusively conducted among women. All studies reported participants’ ages, which ranged from 18 to 85 years old. A number of studies involved students (*n* = 16) with six studies reporting specifically (exclusively) on the association between chronotype and dietary habits among dietetic and nutrition students (*n* = 3), law students (*n* = 1), medical students (*n* = 1), and first-year students (*n* = 1). Three studies were carried out among type two diabetes patients, while one study was conducted among post-bariatric patients and among nutrition outpatients, respectively.

### 3.1. Chronotype Assessment

Circadian rhythm can be measured through physiological markers such as melatonin, cortisol, and body core temperature [50]. The best method to measure circadian rhythm is by measuring the onset of melatonin secretion, which reflects the central circadian clock timing or suprachiasmatic nucleus [51]. However, the melatonin assay is expensive and labour-intensive with the further requirement of participants’ considerable cooperation [52]. The circadian rhythm phase can also be estimated through behavioural patterns such as the sleep–wake cycle, of which the timing varies between individuals, and is known as chronotype [5]. Chronotype or circadian typology is the expression of an individual’s diurnal preferences in performing various biological and psychological traits including the timing of sleep and wakefulness [53]. Individuals who perform daily activities optimally during morning hours, as well as those who sleep and wake up early are known as morning-type or having a morningness preference. In contrast, those who ideally perform activities in the evening hours, sleep and wake up late are recognised as evening-type or having an eveningness preference. The intermediate type refers to individuals who can be either morning or evening type. Chronotype was shown to be correlated with the physiological marker of circadian rhythm, the dim light melatonin onset (DLMO) [52,54]. Hence, this review only included studies that measured chronotype as circadian rhythm proxy. Table 2 shows the type of chronotype assessment and their distribution among included studies. Most included studies measured chronotype via a morningness–eveningness questionnaire (MEQ) by Horne, & Ostberg (1976) (*n* = 17) and three studies used the shortened version of MEQ [9]. Four studies determined chronotype by quantifying the midpoint of sleep [4]. Two studies from Japan used the Japanese version of morningness-eveningness questionnaire [55] specific to the shift-worker population, and two studies used the composite scale of morningness (CSM) [56].

Horne, & Ostberg (1976) initially categorised chronotypes according to five categories, namely definitely morning, moderately morning, intermediate, moderately evening, and definitely evening type. However, a majority of the included studies narrowed down the outcomes into three main categories, namely morning (M-type), intermediate/neutral (I-type), and evening type (E-type). Three of the studies designed cut-off MEQ scoring based on the median score of the respective population studied [30,42,43]. Some studies analysed the chronotype as a continuous variable, whereby the lower the MEQ, the higher the degree of eveningness, and vice versa. Studies that determined chronotype from the midpoint of sleep also presented the outcome as a continuous variable whereby the latest midpoint of sleep indicates a preference towards eveningness and vice versa. All measurements of chronotype reported are well validated and were employed in numerous studies of the human biological clock.

### 3.2. Dietary Assessment

A wide variety of dietary assessment methods were reported in the included studies. Only 19 of the included studies used an established/standardised dietary assessment, for example, validated diet history questionnaire (DHQ) or food frequency questionnaire (FFQ), dietary recall, food record, or food diary. The majority of these studies reported alcohol and caffeine consumption solely based on a questionnaire (yes or no intake) and not the amount of the food intake. Food group was reported as gram/1000 kcal, gram/day, serving/day or gram.

### 3.3. The Association between Chronotype and Dietary Habit

#### 3.3.1. Eating behavior

The most reported association between eating behaviour and chronotype was in terms of meal timing, as found in five out of 12 studies [11,20,26,36,41] (Table 3). A later chronotype (eveningness) was significantly associated with a delay in meal timing. Another six studies [19,21,31,35,40,45] revealed that evening-type individuals had a significantly later meal timing compared to the morning-types. One study also reported a similar meal timing pattern among evening-type individuals with no significant difference between the chronotypes [23]. The majority of the studies also investigated the association between chronotype and timing of breakfast (*n* = 9), lunch (*n* = 8), or dinner (*n* = 6). Silva et al. (2016) was the only study that reported a coefficient of correlation (*r*) whereby a later midpoint sleep time on free days (MSF) was significantly associated with later breakfast (*r* = 0.24) and lunch (*r* = 0.19) times, in spite of a weak correlation [36].

The next most commonly reported association between chronotype and eating behaviour was breakfast consumption. Later chronotype was significantly related to breakfast skippers in three studies, but none of the studies reported *r* value [11,12,36]. Nevertheless, another three studies proved that breakfast skippers were mostly among evening-type individuals compared to morning-type. About 38% of evening-type subjects (vs. 5% of morning-types) missed their breakfast in one study. [23]. On the other hand, Toktas et al. (2018) noted that ~60% of evening-type individuals were skipping breakfast, which is two-fold higher than morning-type individuals who skipped breakfast (33%) [42]. Teixeira, Mota & Crispim (2018) postulated that evening-type individuals were 1.7 times more likely to skip breakfast than morning-types (CI 95%: 1.1–2.9, *p* = 0.02) [40]. In addition, two other studies also found that morning-type individuals regularly ate breakfast, unlike evening-types [26,30].

Night eating was also one of the eating behaviours frequently assessed in the included studies. It was found that individuals classified as evening-type had regular night meals [23], consumed more calories during dinner [34], and before bedtime [37] in contrast to the morning-type individuals. A significant association between chronotype and night eating was observed in Lucassen et al. (2013) where a later chronotype was related to the intake of more calories after 20:00 [31]. These findings were aligned with Reutrakul et al. (2013) who found that a later chronotype significantly consumed more calories during dinner [20]. Moreover, a study using a night eating questionnaire to assess night eating syndrome (NES) discovered a moderate negative correlation between chronotype and NES score (*r* = −0.24, *p* = 0.015), demonstrating that a higher degree of eveningness causes more prominent NES in the study population [28]. Supplementing this evidence, a recent work also replicated similar finding [49].

A later chronotype was also associated with a longer eating duration and frequent TV watching during meals [11]. Eveningness was also significantly related to other eating behaviours such as binge eating [28], emotional eating [41], lower dietary restraint, and higher disinhibition [25]. Nonetheless, one study reported that no significant association between chronotype and the craving of high calorie foods [29]. Another study also found no significant difference in the total number of meals taken per day and portion size between morning and evening types [31]. Interestingly, there were contrasting findings from an assessment of hunger between two types of chronotype. Meule et al. (2012) failed to identify any significant differences in hunger between morning and evening types [30]. However, one study found that morning-type individuals were negatively associated to perceive hunger (*r* = −0.137, *p* = 0.009), with a weak correlation seen [25].

#### 3.3.2. Macro and Micro-Nutrients

The association between chronotype and energy intake was reported in 16 studies and the majority of them found no association (*n* = 9) [10,11,13,20,27,39,43,47,48] or significant differences (*n* = 4) [19,31,34,35] between energy intake among chronotypes (Table 3). However, Mota et al. (2016) found a significant moderate negative association between chronotype scores and energy intake (*B* = −0.28, *p* = 0.02) demonstrating that the greater the eveningness in an individual, the greater the consumption of calories [33]. Furthermore, Toktas et al. (2018) also discovered that evening-type men consumed significantly more energy than morning-type men (2450 kcal ± 625 vs. 1723 kcal ± 543, *p* < 0.001) [42]. However, Teixeira et al. (2018) showed that intermediate-type individuals consumed the most energy compared to evening and morning type individuals (1734 kcal vs. 1690 kcal vs. 1552 kcal, *p* = 0.07), although the morning type had a lower energy intake than the evening type [40].

Out of the 11 studies that were reviewed, the association between carbohydrate intake and chronotype were not significant according to six selected studies [27,31,35,39,40,43]. However, two studies found a significant association between these two variables, although only one study reported the coefficient value [11,33]. Mota et al. (2016) revealed that eveningness was negatively related to the consumption of more carbohydrates (*B* = −0.26, *p* = 0.03) [33]. In contrast, another two studies agreed that evening types consumed significantly more carbohydrate than morning types [34,42]. Only one study found a positive association between morningness and carbohydrate intake [13].

The association between chronotype and protein intake was found in seven studies out of eleven that investigated the connection. Four of the studies reported a significant positive association between morningness and protein intake [11,13,27,39]. Toktas et al. (2018) stated that morning types consumed significantly more protein than evening types [42]. In contrast, eveningness was found to be positively associated with more protein consumption by one study [33]. Munoz et al. (2017) also discovered that normal weight evening types consumed significantly more protein than morning types [34]. Four studies failed to detect any significant association between the variables [31,35,40,43].

There were twelve studies that investigated the association between chronotype and fats intake. The majority of these studies (*n* = 9) however found no significant association between these two variables [10,27,31,33,34,35,39,40,43]. Two studies found that a later chronotype was significantly associated with a greater fat intake [11,13]. Toktas et al. (2018) also revealed that evening types consumed significantly more fat than morning types [42].

Micronutrients were not investigated as vastly compared to macronutrients among the included studies. The association between fibre intake and chronotype were highlighted in four studies [13,39,40,42] and only one study successfully detected a significant association whereby morningness was significantly related to more fibre intake [13]. On the other hand, five studies explored the relationship between cholesterol intake and chronotype [11,27,33,40,42]. Two studies found that eveningness was related to a greater cholesterol intake [11,33], while the other three studies reported no significant difference. Two studies analysed sucrose intake and both discovered similar findings; whereby eveningness was significantly associated with a greater sucrose intake [13,39].

#### 3.3.3. Specific food group

There were eight studies that investigated the association between chronotype and grains (Table 4) [10,11,13,27,33,34,36,48]. From that identified number of studies, only three found that evening types had a significant association with a lower grain intake [10,11,13], while other studies found no significant association. However, one study found that an increase in total grain intake was associated with the eveningness preference [48]. Sato-Mito, Sasaki et al. (2011) studied the association between chronotype and legume consumption, and found an inverse association between late chronotype and legume intake [11]. However, two studies found no significant association between these two variables [33,48].

Among seven studies that explored the relationship between chronotype and meat intake, only two studies detected a significant positive association between meat intake and late chronotype [11,36], while the others found no significant association [10,13,27,33,48]. A significant inverse association between late chronotype and fish intake was reported among several studies [11,27,48]. Interestingly, Maukonen et al. (2016) found a significant association between fish intake and chronotype among female participants alone, whereby evening type women were associated with lower fish intake, and such an association was not significant among men [10]. The association between chronotype and dairy product intake was only observed by Sato-Mito, Sasaki et al. (2011) where later midpoint of sleep was related to lower dairy product intake, while other studies (*n* = 7) found no association [10,13,27,33,34,36,48].

For fruit intake, six studies failed to detect any significant association between chronotype and intake [10,11,13,27,33,36]. Nonetheless, a recent study discovered that eveningness was associated with a lower total fruit intake [48]. Furthermore, evening types reported the consumption of significantly fewer fruits than morning types [8,34,43].

A number of research studies also investigated the association between chronotype and vegetables (*n* = 10). The majority of these studies discovered that eveningness was significantly associated with low vegetables intake [11,13,27]. Furthermore, Patterson et al. (2016) and Yoshizaki et al. (2018) discovered that evening types had a significantly lower vegetable intake compared to the morning types [8,43]. In contrast, Mota et. al. (2016) revealed that evening types among medical residents were significantly associated with a greater vegetable intake whilst other studies (*n* = 4) found no significant association [10,34,36,48].

Several selected studies defined sweets in a category that included sugar sweetened beverages, confectionary, or chocolate intake (*n* = 6). From the total number of studies found, four studies in particular reported that the intake of sweet foods or beverages was significantly associated with eveningness [11,13,33,44], although two studies did not detect any significant association [27,36]. Five studies examined the association between chronotype and fats intake and two discovered significant differences. Sato-Mito, Sasaki et al. (2011) found the latest midpoint of sleep was significantly related to more fat intake [11]. This finding was supported by Munoz et al. (2017) [34]. By contrast, Mota et al. (2016) discovered that the greater the chronotype score (towards morningness), the higher the fat intake among medical residents [33].

Three studies found that evening types consumed significantly more caffeine than morning types [23,37,43]. A significant association between eveningness and caffeine intake was found by Adan (1994) [24]. However, other selected studies detected no significant association between the variables [32,44]. The association between alcohol consumption and chronotype was the most studied component among food groups (*n* = 9). The majority of studies discovered that eveningness was significantly related to a greater alcohol intake (*n* = 6) [10,11,13,24,26,44]. In supporting this statement, another two studies reported that evening types had a significantly greater alcohol intake compared to the morning types [23,34]. Interestingly, one study found no difference in alcohol intake between the chronotype in university students during baseline which was taken earlier in the semester [32]. However, after eight weeks, evening types showed a significant preference for alcohol intake than morning types. Nonetheless, only one study failed to detect any significant difference in alcohol intake between chronotypes [37].

## 4. Discussion

### 4.1. Dietary Behaviour

The aim of the current scoping review was to systematically identify existing literature and to determine the association between chronotypes and various aspects of dietary habits among adults. Table 5 summarises the findings on morning and evening chronotypes’ dietary intake based on the majority significant results of the included studies. An evening chronotype was mostly related to an unhealthy dietary behaviour such as delay in meal timing, excessive food intake during night and skipping breakfast while morning type exhibited the opposite practice. Most importantly, the current review has managed to capture the patterns of each chronotype’s feeding time. Almost all of the studies that explored the aspect of mealtimes discovered similar findings with earlier chronotypes having earlier mealtimes and later chronotype having later mealtimes. Thus, these findings reflect the possibility that the norm of eating time follows a person’s own biological clock despite restrictions to the social clock [30,39,57,58]. Meule et al. (2012) proposed that the biochemical rhythm of appetite-regulating peptides (leptin and ghrelin) might be underlying this behaviour, since the evening chronotype has a more delayed release of ghrelin and leptin than morning chronotypes [30].

The findings from recent research have added to the mounting evidence supporting the connection between chronotype and breakfast [59,60]. A genetic study was conducted among 53 pairs of female twins in order to examine the impact of genetic heritability on meal times [59]. They found that the timing of breakfast (56%) and lunch (38%) was mostly influenced by genetic but not dinner time. In addition, the genetic heritability of waking time was also bigger than sleep time (55% vs. 38%). Hence, this might be the possible underlying mechanism behind the relationship between chronotype and breakfast habits because chronotype was also linked to sleep–wake time [11,27].

Another interesting finding from the latest research is that only the morning chronotype benefited from a higher energy intake during earlier times of the day (within two hours after waking up) and were protected against obesity, with reduced odds of being overweight or obese (*p* = 0.0006). Meanwhile, no significant effect was observed among evening chronotypes [60]. Despite many studies reporting the importance of breakfast [61,62], perhaps not everyone (especially late chronotype) can benefit from its advantages. The composition of meals can also affect or alter the beneficial effects of breakfast [63,64]. Thus, the current review suggests the needs for further research to establish appropriate meal timing and composition that are personalised to an individual’s circadian clock.

The central circadian clock coordinates rhythm in the sleep–wake cycle, thermoregulation, hormonal secretion, metabolism, and feeding behaviour [2,65]. In other words, all of these aspects of our physiology are mapped within a 24 h cycle. Delayed and irregular meal timing can cause circadian misalignment whereby the central circadian clock in SCN is desynchronised from the peripheral clock. The reason for this occurrence is because unlike the central clock where light is the main zeitgeber, feeding is the most potent zeitgeber for the peripheral clock [66,67]. Under normal physiology, feeding–fasting rhythm is triggered by the central clock through sleep-wake cycle [68]. During regular meal timings, circadian clock induces food entrainable oscillator (FEO) to generate food anticipatory activity (FAA) before food ingestion [69]. FAA induce food seeking action by increasing locomotor activity, appetite, body temperature, secretion of digestive enzymes and gastrointestinal motility a few hours before scheduled meal timing, preparing the organism to accept food and thus, optimising the food intake [2]. However, sudden food intake out of the usual meal time still induces peripheral circadian clock (in peripheral tissue and organ) action through acute signalling pathways, but this will not affect the central clock [68].Continuous irregular meal timing might however cause circadian desynchronization between the central and peripheral clock because the central clock in the brain will keep entrained to the day–night cycle while the peripheral clock in organs such as the liver, pancreas, muscles, and white adipose tissue are synchronised to the feeding.

A delay in mealtimes, particularly during dinner, might also cause circadian misalignment because feeding is at risk of shifting towards the resting phase (sleeping time). A human being is a diurnal creature, or in other words, we are meant to do vigorous activities such as working, eating during the day while resting and fasting during night. During the day, the ingestion of food will provide substrates (glucose, amino acid and lipid) to support the metabolic processes in body cells, whereas at night, the body’s storage of energy and substrates will be used to maintain metabolic homeostasis [70]. Hence, food intake during the rest phase is against our internal clock rhythm and is linked to diseases such as obesity, diabetes, and cardiovascular disease. There has been a rise in the number of studies investigating the effects of feeding during the circadian rest phase in animals [71,72,73,74,75]. For instance, shifting a rat’s feeding time to the rest phase (during the day) altered the normal rhythm of resting energy requirement (RER) [71,74,75]. The ad libitum fed rat (control group) showed a decrease in RER during the day, whereas it increased and remained high at night (active phase). In contrast, day-fed rats showed an opposite rhythm whereby RER was high during the day and maintained at the level above 1.0, indicating an increase in de novo lipogenesis, and thus, it may have promoted fat mass deposition [76]. A recent study have proven that even short term feeding during resting phase can induce leptin resistance in mice [73]. These mice were fed ad libitum for two weeks and were then divided into day-fed and night-fed groups for 11 days. Leptin was injected on day six for both groups. The night-fed group demonstrated a reduction in food intake (34%) and body weight (5%), however, leptin injection had no significant effect on day-feed mice implying leptin resistance. Furthermore, the researchers found that day-fed mice had higher triglyceride, free fatty acid, and total cholesterol levels compared to the night-fed mice. Nonetheless, those studies were conducted to simulate extreme shifts of meal timing during the rest phase, which mostly occurs among shift workers. There are a limited number of studies available on the impact of delays in meal timing among individuals with evening chronotypes. The current review noted that the delay in dinner or night meals among evening chronotypes was less severe compared to shift workers (range from 19:19 to 21:31). Nonetheless, evening chronotypes remain exposed to a milder form of chronodisruption (regards irregular and delay meal timing) [77]. Thus, more studies are needed to explore whether this habit has the possibility to cause adverse long-term effects.

The current review also reports in Table 5 that the following dietary behaviour was limitedly assessed by the included studies: food addiction, hunger, eating duration, watching TV during the meal, binge eating and meal skipping. Hence, more evidence is needed to investigate the association between chronotype and these dietary behaviours.

### 4.2. Macro and Micro Nutrients and Food Group

In terms of macronutrient intake, the majority of the included studies agreed that different chronotypes’ intake of energy, carbohydrate, and fat did not significantly differ (Table 5). However, Maukonen et al. (2017) further analysed the nutrient intake based on feeding timing (during morning and at night) and obtained significant results [39]. Evening chronotypes were found to consume less energy by 4%–5% in the morning, and as much as 6%–7% more energy at night compared to morning chronotypes [39]. This finding highlights the differences in the eating patterns between chronotypes in spite of consuming approximately the same amount of energy and nutrients, and also emphasises the importance of including the timing of food ingestion in dietary assessments. Another interesting discovery was that morning chronotypes were positively associated with a greater protein intake while evening types were associated with a greater sucrose intake. The early chronotypes were also linked to having a higher consumption of protein during early times of the day and this was also related to a decrease in the likelihood of being overweight and obese (*p* < 0.03) [60]. Nonetheless, evening chronotypes were linked to detrimental eating habits of sucrose which have notable negative effects on the health such as obesity, diabetes, an increase in cholesterol and blood pressure levels [78,79]. However, the current review cannot conclude the actual amount of sucrose intake by evening chronotypes due to the variations in the reporting of nutrient intake by the included studies. Hence, these findings need careful interpretation because the effects from normal consumptions of sucrose on the health is still undefined [79]. For vitamins and minerals, the current review was unable to establish a robust relationship with chronotypes because these variables were limitedly evaluated in the chosen studies.

The current review also emphasises on data concerning specific food intake by each chronotype because a single food group can be a major source of a variety of macro and micro-nutrients. For instance, grains constitute a notable supply of carbohydrates and an assortment of nutrients such as fibre, vitamin B, zinc, iron, and magnesium [80]. Animal sources constitute a high quality source of protein that supplies other essential nutrients. For instance, fish provides omega-3 fatty acids [81] while meat fulfils bodily needs of amino acid, iron, zinc, and vitamin B [82]. Hence, it is also important to capture the type of food intake, as well as to translate the information into nutritional value. The authors summarised that evening chronotype are usually engaged with unhealthy eating practices with lower vegetables consumption and higher intake of sweet food/beverages, caffeine, and alcohol (Table 5). On the other hand, no difference was noted between chronotypes for the consumption of legumes, meat, fish, and dairy products. No sufficient evidence was available to conclude an association between grains, fruits or fats, and oil with chronotypes because there was a similar number of studies that showed both the presence and absence of an association.

Importantly, the current review has detected the connection between nutrients and specific food group intake of chronotypes. Even though evening types are mostly linked to low vegetables intake but quite a number of studies have reported that they also consumed similar amounts of fruits as morning types. The current review proposed that this might be the reason for the similar fibre intake between the two chronotype groups. Similar outcomes reported for grains, fats, and oil intake reflected the similar nutrient intake of carbohydrate and fat respectively. There is a need for more studies to allow for a refined analysis of the grains group (whole grains and total grains intake) in a future systematic review. Perhaps, there is a possible significant difference in terms of carbohydrate quality between the chronotypes.

Evening chronotypes were reported to consume more alcohol and caffeine, which is widely recognised for its stimulant properties that can boost wakefulness and concentration by increasing brain activity [83,84,85]. However, many other studies have demonstrated how an excessive intake of alcohol and caffeine can result in reduced sleep duration and quality, increased fatigue and daytime sleepiness, and delayed sleep onset [85,86,87,88,89,90]. According to Galli et al. (2013), each alcoholic drink (contain 14g of alcohol) can reduce sleep duration by 30 min. In addition, the history of binge alcoholic drinking during the adolescent age was found to be linked to a diminishment of sleep quality during the adult stage among Mexican Americans and American Indians [87]. Fucito et al. (2018) also discovered that reduction in sleep duration influences a greater number of alcoholic drinks consumed on the following day among young adults. The mechanism underlying this association is still unclear, but it is proposed that both alcohol and caffeine intakes can delay the onset on nocturnal melatonin release, thus suppressing sleepiness [83,91]. Interestingly, some studies revealed how the consumption of alcohol and caffeine can increase the odds of later chronotype [90,92]. It was postulated that evening chronotypes have more chances to engage with ‘evening habits’ (drinking) due to a delay in sleep time compared to morning chronotypes, who sleep earlier [93]. Complementing that statement, the included studies also revealed that evening chronotypes significantly consumed the most number of alcohol beverages after 20:00 than morning and intermediate chronotypes [39]. Taking these reported findings into consideration, the present review emphasises the importance of appropriate timing for specific food intake.

### 4.3. In Relation to Obesity and Weight Loss

Unhealthy eating practices is one of the risk factors linked to the development of obesity. A systematic review concluded that a diet consisting of high dietary fibre intake predicts less weight gain, whereas high consumption of sweets and desserts predicts greater weight gain [94]. Besides, another study also reported that excessive intake of sugary beverages, and a low intake of fruit and vegetables are all linked to the increase of adiposity [95]. Aforementioned in the review, the majority of the selected studies agreed that evening chronotypes displayed unhealthy eating practices which can raise risks of obesity. Most studies included in this review also reported information on weight or BMI, and most of the cross-sectional studies have shown no difference in weight markers between morning and evening chronotypes (Table 1). This might be due to many of these studies being conducted among university students, and this age group population is young and mostly healthy; making them more resistant towards the effects of misalignment in sleep/wake timing [96]. Studies that were conducted among the same population would usually report the same outcome where the majority of their respondents had a healthy weight range [97,98]. Furthermore, a cross-sectional study captures the weight parameter at one time only.

In contrast, a follow-up study [32] discovered that although there were no association detected between chronotypes and weight during baseline, the researcher found that later chronotypes were significantly linked to higher weight and BMI after eight weeks. Moreover, a prospective cohort study among six-years post-bariatric surgery patients demonstrated that evening types had a greater weight pre-surgery and experienced less excess weight loss compared to the morning types [35]. Although both chronotypes had almost the same energy intake, evening types had later lunch and dinner timing compared to the morning types, aligning with the finding of the current review. Consistent with this finding, Garaulet et al. (2013) also found that those who had lunch later were exhibited slower weight loss progress in their intervention despite having the same calorie intake and dietary composition as early lunch eaters; with majority of late lunch eaters being evening chronotypes [21]. Other intervention studies also found that morning types had better weight loss outcome compared to evening types [34,99]. In fact, recent findings discovered that circadian misalignment can also increase obesity risk among morning types [96]. A short sleep duration (less than 6 h), reduced sleep efficiency (less than 85%) or staying awake after sleep timing (more than 60 min) can increase body fat percentage, waist:hip ratio and waist:height ratio. Lastly, the impact of night meals differed according to the chronotypes in that late chronotypes acquired an increased risk of being overweight or obese yet no significant effects were found affecting early chronotypes [60].

With this in consideration, chronotypes might play an important role in the mechanism of weight gain and obesity in relation to dietary habits. Disturbance in active (feeding) and resting (sleeping) phase activities have become risk factors for the development of obesity and can hamper weight loss effectiveness. More studies are needed to determine the relationship between chronotype and dietary habits especially in relation to meal timing and obesity. The strength and limitations of this review should be carefully considered. This review has compiled a database of studies on the association between chronotype and three major dietary aspects, namely dietary behaviour, nutrient intake, and specific food group intake. This step is crucial in identifying each chronotype’s dietary aspects to develop a specific chrono-nutrition intervention for weight management or any dietary related health interventions. There are several limitations in the current scoping review. First, most of the included studies were cross-sectional design due to insufficient evidence on clinical trial in this area. Secondly, the current review generally discusses individuals without a sleep disorder because chronotype is affected by sleep and wake timing. However, it is important to highlight that there are other factors that influence chronotype, such as age, gender, latitude, work schedule, and social engagement [100]. Hence, future systematic reviews could more specific control for these modifiable and non-modifiable factors that affect chronotype. Lastly, the chronotype and the dietary assessment applied in the included studies varied widely, and thus no statistical analysis was conducted to conclude this scoping review. The current review highlights the need for more randomised clinical trial studies and thorough dietary assessments and diet quality to further clarify the link between chronotype and dietary aspects to enhance the effectiveness of weight management intervention.

## 5. Conclusions

In conclusion, the circadian preference towards eveningness is associated with a delay in meal timing, the breakfast skipping habit, engagement with excessive consumption during night time, lower protein and vegetables intake, as well as increased sucrose, sweets, caffeine, and alcohol intake. A limited number of studies have also shown that the aforementioned chronotype was related to a lower intake of grains and fruits. Despite that, the current review has discovered that both morning and evening chronotypes relatively consumed the same number of calories, amount of carbohydrates, fat, cholesterol, fibre, legumes, meat, fish and dairy products. Nonetheless, more aspects of dietary behaviours, micronutrients, and other food group intakes between chronotypes remain uncertain, and more studies are required to elucidate the relationship between chronotype and body weight in the long term.

## Figures and Tables

**Table 1:** Characteristics of the selected studies.

Reference	Country	Study Design	N	% Female	Age (Year)	Participants	Weight Status (If Reported)
[23]	Japan	Cross-sectional	1459	37.5	Mean: 19.5	University students	-
[24]	Spain	Cross-sectional	537	52.1%	Range: 21–30	University students and lecturers	-
[25]	German	Cross-sectional	335	58.8	Mean: 23.15 Range: 17–42	University students	Mean BMI: 22.93 ± 3.41 kg/m^2^
[26]	Japan	Cross-sectional	800	100.0	Mean: 19.26 ± 1.33 Range: 18–29	Students	-
[11]	Japan	Cross-sectional	3304	100.0	Mean: 18.1 ± 0.3 Range: 18–20	Dietetics Students	Mean BMI: 20.9 ± 2.8 kg/m^2^
[27]	Japan	Cross-sectional	112	100.0	Range: 19–36	Dietetics students	Mean BMI: 19.9 ±1.8 kg/m^2^
[28]	Brazil	Cross-sectional	100	77.0	Mean: 39.5 ± 11.7 Range: 18–65	Outpatient nutrition clinic	Mean BMI: 26.8 ± 4.02 kg/m^2^
[13]	Finland	Cross-sectional	4493	67.0	Mean: 51.9 ± 0.2 Range: 25–74	Population based study	Mean BMI: 26.8 ± 0.2 kg/m^2^
[29]	Malaysia	Cross-sectional	1118	56.0	Mean: 20.06 ± 1.53 Range: 18–27	University students	-
[30]	Germany	Cross-sectional	66		Mean: 23.08 ± 2.68	University students	Mean BMI: 22.22 ± 4.48 kg/m^2^
[21]	Spain	Observational Intervention study	420	49.5	Mean: 42 ± 11	Outpatient of nutrition clinic	Mean BMI: 31.4 ± 5.4 kg/m^2^
[31]	USA	Prospective, randomized controlled study	126	77.0	Range: 18–50	Population based study	Range BMI: 30–55 kg/m^2^
[12]	USA	Cross-sectional	194	69.6	Range: 18–85	Type 2 diabetes patients	Mean BMI: 35.6 ± 8.3 kg/m^2^
[20]	USA	Cross-sectional	194	70.0	Range: 18–85	Type 2 diabetes patients	Mean BMI: 35.6 ± 8.3 kg/m^2^
[32]	USA	Prospective study	137	58.0	Range: 18.25 ± 0.56	University freshmen	Mean BMI: 21.99 ± 3.24 kg/m^2^
[10]	Finland	Cross-sectional	4421	54.0	Range: 25–74	Population based study	-
[33]	Brazil	Cross-sectional	72	72.0	Mean: 29.2 ± 2.0	Medical residents	Mean BMI: 22.9 ± 3.4 kg/m^2^
[34]	Spain	Cross sectional & Interventional longitudinal study	400,171 finished follow up	Not stated	Range: 30–60	University staff	Mean BMI: - M-type: 28.2 ± 5.9 kg/m^2^ - E-type: 28.5 ± 6.1 kg/m^2^
[8]	USA	Cross-sectional	439,933	56.0	Mean: 56.5 ± 8.1 Range: 40–69	Population based study	Mean BMI: 27.4 ± 4.8 kg/m^2^
[35]	Spain	Prospective cohort study	252	79.0	Age: 52 ± 11	Post bariatric surgery patient	Mean BMI: 46.4 ± 6.0 kg/m^2^
[36]	Brazil	Cross-sectional	204	55.0	Mean: 21.6 ± 3.9 Range: 18–39	Undergraduate of Law School	Mean BMI: 22.8 ± 3.2 kg/m^2^
[37]	Korea	Cross-sectional	2976	51.0	Mean: 58.02 ± 7.05 Range: 49–79	Population based study	Mean BMI: - M-type: 25.0 ± 6.8 kg/m^2^ - I-type: 24.8 ± 5.3 kg/m^2^ - E-type: 24.8 ± 3.4 kg/m^2^
[38]	Japan	Cross-sectional	218	100.0	Range: 21–63	Nurses (day and rotating shift)	Mean BMI: 21.7 kg/m^2^
[39]	Finland	Cross-sectional	1854	54.0%	Range: 25–74	Population based study	Mean BMI: - M-type: 27.1 ± 0.2 kg/m^2^ - I-type: 26.7 ± 0.2 kg/m^2^ - E-type: 27.6 ± 0.3 kg/m^2^
[40]	Brazil	Cross-sectional	721	67.7	Above 18 years old	Undergraduate student	Mean BMI: - M-type: 22.6 ± 3.2 kg/m^2^ - I-type: 22.3 ± 3.8 kg/m^2^ - E-type: 22.2 ± 3.6 kg/m^2^
[41]	Spain	Cross-sectional	2126	81.0	Mean: 40 ± 13	Overweight and obese population	Mean BMI: 31 ± 5 kg/m^2^
[19]	Thailand	Cross-sectional	210	60.0	Mean: 58.6 ± 11	Type 2 diabetes	Mean BMI: 28.4 ± 4.8 kg/m^2^
[42]	Turkey	Cross-sectional	142	43.0	Mean: 21.83 ± 2.37	University student	Mean BMI: - M-type: 22.7 ± 2.8 kg/m^2^ - I-type: 22.3 ± 2.9 kg/m^2^ - E-type: 22.8 ± 3.2 kg/m^2^
[43]	Japan	Cross-sectional	2559	100.0	Range: 20–59	Nurses (day and rotating shift workers)	Mean BMI: - day worker: 21.2 ± 2.7 kg/m^2^ - shift worker: 21.6 ± 3.2 kg/m^2^
[44]	China	Cross-sectional	977	57.7	Mean: 20.06 ± 1.25	University undergraduates	BMI: - 20% underweight - 73% normal weight - 7% overweight or obese
[45]	India	Cross-sectional	203	35.5	Mean: 18.34	University student (medical)	Mean BMI: - E-type: 23.06 ± 2.45 kg/m^2^ - I-type: 22.50 ± 2.15 kg/m^2^ - M-type: 21.89 ± 2.27 kg/m^2^
[46]	Turkey	Cross-sectional	383	60.1	Mean: 21.1 ± 0.1 Range: 17–37 years	University students	Mean BMI: 22.25 ± 3.19 kg/m^2^
[47]	Finland	Follow-up, 7 years	Baseline: 5024 Follow-up: 1097	54.0	Range: 25–74	Population based study	-
[48]	Brazil	Cross-sectional	100	100	27.3 ± 5.7	Pregnant women	-
[49]	Turkey	Cross-sectional	1323	65.8	Mean: 20.83 ± 1.98 Range: 16–33	University students	Mean BMI: 21.96 ± 3.03 kg/m^2^

BMI, body mass index; M-type, morning chronotype; I-type, intermediate chronotype; E-type, evening chronotype.

**Table 2:** The assessment and distribution of chronotypes.

Reference	Chronotype	
	Assessment	Distribution
[23]	MEQ	M-type: 110 (7.5%) E-type: 339 (23%)
[24]	MEQ	M-type: 106 (20%) E-type: 108 (20%) Neither: 323 (60%)
[25]	CSM	M-type: 14 (4.2%) E-type: 51 (15.2%) I-type: 270 (80.6%)
[26]	ME	Mean ME score: 16.07 (3.53)
[11]	Midpoint of sleep	Quintile 1 (earliest midpoint of sleep): 534 (16%) Quintile 5 (latest midpoint of sleep): 601 (18%)
[27]	MEQ Midpoint of sleep	High MEQ score: 37 (33%) Low MEQ score: 37 (33%) Early midpoint of sleep tertile: 40 (36%) Late midpoint of sleep tertile: 37 (33%)
[28]	MEQ	Mean MEQ: 52.4 ± 14.0
[13]	Shortened MEQ	Quintile 5 (extreme M-type): 22% Quintile 1 (extreme E-type): 18%
[29]	MEQ	Moderate E-type: 18.2% Definite E-type: 81.6% I-type: 0.2%
[30]	MEQ	M-type: 35 (53%) E-type: 31 (47%)
[21]	MEQ	Not stated
[31]	MEQ	M-type: 67% E-type: 39%
[12]	MSF	Mean MSF of breakfast skippers: 4:34 (2.18)
[20]	MSF	Mean MSF: 3.29 (1.46)
[32]	Reduced MEQ	M + I-type:72 (53%) E-type: 64 (47%)
[10]	MEQ	M-type: 37% E-type: 28%
[33]	MEQ	M-type: 36% E-type: 14% I-type: 50%
[34]	MEQ	M-type: 47% E-type: 53%
[8]	Self-report chronotype	Early type: 27% Intermediate-early type: 36% Intermediate-late type: 28% Late type: 9%
[35]	MEQ	M-type: 14.3% E-type: 26.2% Neutral type: 59.5%
[36]	MSF	Mean MSF: 5.40 (1.48)
[37]	MEQ	M-type: 38.2% E-type: 4.9% I-type: 56.9%
[38]	ME	Day workers: 20.8 ± 3.3 Shift workers: 17.1 ± 4.0 (Lower scores, more eveningness)
[39]	MEQ	M-type: 49% E-type: 12% I-type: 39%
[40]	MEQ	M-type: 21% E-type: 17% I-type: 62%
[41]	MEQ Dichotomous based on median score of population; 53	M-type: 52% E-type: 48%
[19]	CSM	E-type (CSM < 45): 46% M-type (CSM ≥ 45): 54%
[42]	MEQ	M-type: 13% E-type: 24% I-type: 63%
[43]	MEQ Higher scores and tertiles (T) indicate a tendency towards morningness.	MEQ score in; (a) Day worker: T1: 34-53 (36%) T2: 54-59 (33%) T3: 60-76 (31%) (b) Shift worker: T1: 25-49 (35%) T2: 50-56 (36%) T3: 57-77 (29%)
[44]	MSF	Mean MSF: 4:41(1:06)
[45]	MEQ	M-type: 21% E-type: 36% I-type: 43%
[46]	MEQ	M-type: 73 (19.1%) E-type: 54 (14.1%) I-type: 256 (66.8%)
[47]	MEQ	M-type: 50.3% E-type: 10.2% I-type: 39.5%
[48]	MSF	M-type: 42% E-type: 22% I-type: 36%
[49]	MEQ	M-type: 15.4% E-type: 14.6% Mix-type: 70.0%

ME, Morningness–Eveningness (Torsvall & Akerstedt 1980); MEQ, morningness–eveningness questionnaire (Horne and Ostberg 1976); MCTQ, Munich chronotype questionnaire; CSM, composite scale of morningness; MSF, midpoint of sleep on free days.

**Table 3:** The association of chronotype with dietary behaviour, nutrient intake and other health status.

Reference	Measure of Dietary Pattern	The Association of Chronotype		
		Dietary Behaviour	Nutrient Intake	Other Health Status
[23]	Life Habits Inventory	E-type significantly had frequent night meal (χ = 65.63, *p* < 0.001) compared to M-type. 34.8% of E-type skipped breakfast than M-type, 5.5%. No significant different in meal timing; breakfast, lunch and dinner between chronotypes.	-	-
[25]	Three-factor eating questionnaire (TFEQ)	Positive significant association between M-type and dietary restraint (*r* = 0.136, *p* = 0.013). Negative significant association between M-type with disinhibition (breaking dietary restraint and overeating) (*r* = −0.151, *p* = 0.006) and perceived hunger (*r* = −0.137, *p* = 0.009).	-	-
[26]	Questionnaire on life habits Examination of eating habits	Higher chronotype score (towards morningness) significantly related to regular breakfast eater (*χ*^2^ = 74.55, *p* < 0.001) and earlier mealtime for breakfast (*χ*^2^ = 88.94, *p* < 0.001).	-	-
[21]	Seven-day dietary record	E-type significantly were more late lunch eaters (after 15:00) than M-type (*p* = 0.032).	-	No significant association between weight loss (%) and MEQ score (*p* = 0.456).
[11]	Lifestyle & dietary behaviour questionnaire Diet history questionnaire (DHQ)	Quintile 5 (towards eveningness) compared to Quintile 1 (towards morningness) significantly: delay in meal timing during breakfast (9:00 ± 0.02 vs. 6:35 ± 0.02, *p* < 0.001), lunch (12:42 ± 0.02 vs. 12:20 ± 0.02, *p* < 0.001) and dinner (19:19 ± 0.05 vs. 18:51 ± 0.06, *p <* 0.01) skipped breakfast (1.91 ± 0.07 vs. 0.66 ± 0.07 times/week, *p <* 0.01) takes longer time to eat during breakfast (19.03 ± 0.18 vs. 17.38 ± 0.02 min.sec, *p <* 0.01), lunch (25.29 ± 0.19 vs. 21.50 ± 0.23 min.sec, *p <* 0.001) and dinner (32.29 ± 0.26 vs. 28.45 ± 0.31 min.sec, *p <* 0.001) watching TV during meals; breakfast (3.55 ± 0.07 vs. 3.27 ± 0.08 times/week, *p <* 0.05), lunch (3.24 ± 0.07 vs. 1.25 ± 0.08 times/week, *p <* 0.001) and dinner (3.90 ± 0.06 vs. 3.63 ± 0.07 times/week, *p <* 0.05).	Latest midpoint of sleep (towards eveningness) significantly associated with: consumptions of less % of energy from carbohydrate and protein and intake pf cholesterol, potassium, calcium, magnesium, iron, zinc, vitamin A, vitamin D, thiamine, riboflavin, vitamin B6 and folate. consumption of more % of energy from fat. No association between midpoint of sleep with total energy intake/day.	No significant association between BMI and midpoint of sleep (*p* = 0.30).
[29]	Craving of High-calorie foods questionnaire	Not significant relationship between chronotype and high calorie food craving (*r* = 0.003, *p* = 0.917)	-	No significant association between BMI and MEQ score (*r* = 0.043, *p* = 0.152).
[27]	Brief diet history questionnaire (BDHQ)	-	A lower chronotype score (towards eveningness) was significantly associated with less energy from protein and intake of calcium, magnesium, zinc, vitamin D, riboflavin, vitamin B6 and folate. No association between chronotype score with total energy intake/day, carbohydrate, fats and other micronutrients. Latest midpoint of sleep (towards eveningness) significantly associated with lower energy from protein and intake of cholesterol, potassium, calcium, magnesium, zinc, vitamin D, riboflavin, vitamin B6 and vitamin B12. No association between midpoint of sleep with total energy intake/day, carbohydrate, fats and other micronutrients.	No significant association between BMI and midpoint of sleep (*p* = 0.67) and MEQ score (*p* = 0.78).
[12]	24 h diet recall	Later MSF (towards eveningness) significantly were breakfast skippers (*p* = 0.002).	-	-
[30]	Food craving questionnaires	Significantly more M-type; 91.2% had breakfast compared to E-type; 46.4% (*p* < 0.001). There is no significant difference in hunger between morning and evening type.	-	No significant association between BMI and MEQ score (*F* = 0.52, *p* > 0.05).
[28]	Binge eating score (BES), eating attitudes test (EAT) and night eating syndrome (NES)	Lower chronotype score (towards eveningness) was significantly associated with higher binge eating (*r* = −0.33, *p* = 0.001) and night eating syndrome score (*r* = −0.24, *p* = 0.015). In multivariate regression (*r*^2^ = 0.12, *F* = 6.8, *p* = 0.002), only binge eating remained significantly associated with chronotype score (*β* = −0.25, *p* = 0.028) No correlation was found between EAT and chronotype.	-	No significant association between BMI and MEQ score (*r* = −0.101, *p* = 0.319).
[31]	Three-day food record	E-type delay in breakfast time during working (8:38 ± 1:52 vs. 7:17 ± 1:31, *p <* 0.001) and non-working days (9:52 ± 2:32 vs. 8:56 ± 2:30, *p* = 0.075) compared to M-type. Lower chronotype score (towards eveningness) related to consumptions of more calories after 20:00 (*β* = 0.459, *p <* 0.001). No significant differences in portion size and number of eating occasion between chronotypes.	No significant differences in total energy intake between chronotype. Meals consumed after 20:00 contained less carbohydrate (49 ± 16% vs. 53 ± 10%, *p* = 0.021) and protein (12 ± 7% vs. 14 ± 4%, *p* = 0.006) and more fat (34 ± 14% vs. 32 ± 7%, *p* = 0.069). However, there was no significant difference between chronotype in these macronutrients.	No significant difference in weight between M and E-type. However, chronotype score (moving from morningness to eveningness score) was associate with increase in BMI (*r*^2^ = 0.057, *p* = 0.048), larger neck circumference (*r*^2^ = 0.488, *p* = 0.028) and lower HDL-C levels (*r*^2^ = 0.095, *p* = 0.026).
[37]	Sleep interfering behaviour scale	E-type significantly ate heavy meal before bedtime compared to I-type and M-type (*p <* 0.001).	-	No significant difference in BMI between the chronotypes.
[13]	Food frequency questionnaire	-	Quintile 1 significantly consume more energy from alcohol (2.5 vs. 1.8 E%, *p <* 0.001), fat (31.3 vs. 30.8 E%, *p <* 0.001), saturated fatty acid (11.6 vs. 11.5 E%, *p* = 0.002) and sucrose (9.8 vs. 10.0, *p* = 0.001) compared to Quintile 5. Quintile 5 significantly consume more energy from carbohydrates (49.5 vs. 48.6 E%, *p <* 0.001), protein (17.9 vs. 17.6 E%, *p* = 0.16) and intake of more fibre (32 vs. 30 g, *p <* 0.001), folic acid (426 vs. 412 µg, *p <* 0.001), vitamin D (9.9 vs. 9.5 µg, *p <* 0.001) and sodium (3.9 vs. 3.8 g, *p <* 0.001) compared to Quintile 1. No significant difference between chronotype quintiles in total energy intake, vitamin C, and calcium intake.	No significant association between BMI and chronotype score (*p* = 0.35).
[10]	Food frequency questionnaire	-	In women, a lower chronotype score (towards eveningness) was associated with more % energy intake from fat (*p* < 0.018), adjusted to age. No significant association among men. No significant differences in total daily energy intake between chronotype in both men and women.	In men, there was a positive association between chronotype and BMI (*B* = 0.048, *p* = 0.041). Chronotype modified the association between the healthy diet and body fat % and waist circumference.
[20]	24 h diet recall	MSF Quartile 5 (towards eveningness) compared to MSF Quartile 1 (towards morningness) significantly: delay in meal timing; breakfast (9:48 ± 1:22 vs. 7:47 ± 1:39, *p <* 0.001) and dinner (19:19 ± 1:3 vs. 18:25 ± 1:13, *p* = 0.02) consume more calories during dinner (43 ± 23% vs. 32 ± 20%, *p* = 0.04)) No significant difference in consumptions of calories during breakfast between chronotypes.	No significant differences in total daily energy intake between chronotype.	Later MSF was associated with higher BMI (*p* = 0.03). Later MSF was significantly associated with higher HbA_1c_ (*B* = 0.025, *p* = 0.001); 1 h delay of MSF was associated with an increase in HbA_1c_ of 2.5% from the original level.
[33]	3 days food diary	-	Lower chronotype score (towards eveningness) were significantly negatively associated with consumption of more calories (*β* = −0.28, *p* = 0.02), carbohydrate (*β* = −0.26, *p* = 0.03), protein (*β* = −0.23, *p* = 0.04) and cholesterol (*β* = −0.24, *p* = 0.04).	Chronontype scores not associated with BMI (*B* = −0.01, *p* = 0.98), WC (*B* = 0.09, *p* = 0.41) and weight gain (*B* = −0.1, *p* = 0.48) after the beginning of residency.
[36]	Food frequency questionnaire	Breakfast skippers (12.2%) significantly had later MSF (towards eveningness) (6:19 vs. 5:28, *p* = 0.02) than those who had breakfast. Later MSF significantly positively associated with delay meal timing during breakfast (*r* = 0.24, *p <* 0.001) and lunch (*r* = 0.19, *p <* 0.01)	Later MSF (towards eveningness) was significantly positively associated with greater servings/day of meat (*β* = 0.21, *p* = 0.003), adjusted for age and BMI.	-
[41]	24 h diet recall Eating behaviour score Emotional eating questionnaire	A lower chronotype score (towards eveningness) was significantly associated with delayed meal timing during breakfast (*p <* 0.001), lunch (*p* = 0.002) and dinner (*p* = 0.007). E-type significantly had higher eating behaviour score (larger portion sizes, second rounds and energy rich foods) and emotional eating score compared to M-type (*p <* 0.001).	E-type consume significantly less carbohydrate (193.78 ± 3.18 vs. 204.59 ± 3.07 g, *p* = 0.017) than M-type. There was no significant difference between chronotype in total energy, protein, and fats intake.	Lower chronotype score (towards eveningness) was significantly associated with higher BMI (*p* = 0.032). and triglyceride (*p* = 0.006). and lower HDL-cholesterol (*p* = 0.001).
[35]	4 day food records	E-type significantly delayed mealtime during lunch (14:19 vs. 14:04, *p* = 0.017) and dinner (21:31 vs. 21:06, *p <* 0.001) compared to M-type.	No significant differences in total daily energy intake and macronutrients (carbohydrate, protein and fat) during baseline and follow up between chronotype.	E-type had more body weight (126.0 ± 22.3 vs. 119.8 ± 15.9 kg, *p* = 0.020) during pre-bariatric surgery and loss less excess weight loss (EWL) (77.9 ± 23.3 vs. 82.9 ± 22.6%, *p* = 0.041) post-bariatric surgery than M-type. In CLOCK 3111 carrier of risk allele C, E-type significantly were more obese during baseline than M-type (*p* = 0.012).
[45]	Proforma (questionnaire)	There were significantly more E-type (75.34%) had delay in dinner timing (later than 21:00) that I- (41.38%) and M-type (34.88%), *p* < 0.001.	-	E-type had significantly greater BMI than I and M-type (*p* = 0.029).
[34]	Food frequency questionnaire	Among normal weight participants, compared to M-type, E-type significantly consume: fewer calories during lunch (40.1 ± 1.5 vs. 45.5 ± 1.3%, *p* = 0.008) more calories during mid-evening snack (7.0 ± 1.0 vs. 4.6 ± 0.7%, *p* = 0.041) and dinner (32.3 ± 1.9 vs. 27.6 ± 1.5%, *p* = 0.059). Among overweight participants, compared to M-type, E-type consume: less calories during breakfast (12.0 ± 0.4 vs. 14.5 ± 0.5%, *p* = 0.004) and lunch (42.7 ± 0.8 vs. 44.5 ± 0.9%, *p* = 0.01) more calories during mid-morning snack (7.5 ± 0.3 vs. 5.3 ± 0.5%, *p* = 0.021) Among M-type individuals, overweight participants consumed significantly more calories during dinner compared to normal weight participants (31.1 vs. 27.6%, *p <* 0.05). Among E-type, normal weight participants consumed significantly more calories during breakfast compared to overweight participants (14.5 vs. 12.0%, *p <* 0.01)	No significant differences in total daily energy intake, carbohydrate, protein and fat between chronotype among overweight participants. Among normal weight participants, evening type significantly consumed more carbohydrate (*p* = 0.024) and protein (*p* = 0.003) than M-type. No significant difference in total energy and fat intake.	M-type group loses more body weight (−0.75 ± 0.54 vs. −0.60 ± 0.46 kg/week, *p* = 0.153), BMI (-3.30 ± 0.53 vs. −2.63 ± 0.49, *p* = 0.133) and body fat (−5.41 ± 1.98 vs. −5.37 ± 2.3 %, *p* = 0.912) than E-type.
[38]	Eating behaviour questionnaire	Lower chronotype scores significantly negatively associated to higher meal contents (*β* = −0.172, *p* = 0.041) and temporal meal timing (*β* = −0.338, *p* < 0.001). A higher meal contents score represents greater preference towards high-fat diet and sweets. A higher temporal eating score represent greater irregular meal timing and total of meals consumed and delay meal timing.	-	-
[19]	24 h dietary recall	E-type significantly delay meal timing during breakfast (7:30–9:00 vs. 7:00–8:30, *p <* 0.001), lunch (12:00–13:23 vs. 12:00–13:00, *p* = 0.032), dinner (18:00–19:00 vs. 17:30–19:00, *p* = 0.031) and last meal (18:00–19:38 vs. 17:53–19:00, *p* = 0.03) time than M-type. M-type was significantly negatively associated with breakfast time (*β* = −0.614, *p* < 0.001).	There was no significant difference between total energy intake between chronotype.	Greater preference towards eveningness was associated with greater BMI (*B* = −0.141, *p* = 0.019). Mediation analysis shown, M-type was associated with earlier breakfast time and thus lower BMI by 0.37 kg/m^2^ (*B* = −0.365, 95%CI: −0.877, −0.066).
[49]	Yale food addiction scale	Chronotype score (towards eveningness) was significantly negative associated with food addiction (*r* = −0.10, *p* < 0.01).	-	No significant difference in BMI between the chronotypes.
[39]	48 h dietary recalls 3 days food records	-	During weekdays and weekends, E-type significantly consumed: 4%–5% less energy in morning (by 10 am) and, 6%–7% more energy at night (after 20:00) compared to M-type. No significant difference between the chronotype in total daily energy intake on both weekdays and weekends. On weekdays, lower chronotype score (towards eveningness) was significantly associated with: Less carbohydrate, protein, fat, fibre and saturated fatty acids and more sucrose in the morning (by 10 am). More carbohydrate, fat, saturated fatty acids, and sucrose at night (after 20:00). On weekends, a lower chronotype score (towards eveningness) was significantly associated with: Less carbohydrate, protein, fat, saturated fatty acids and fibre in the morning (by 10:00) More carbohydrate, fat, saturated fatty acids and sucrose at night (after 20:00).	No significant difference in BMI between the chronotype.
[40]	24 h diet recall	E-type significantly had delay meal timing during breakfast (8:00 ± 1.2 vs. 7:20 ± 1.1, *p <* 0.001) and lunch (12:38 ± 1.00 vs. 12:13 ± 0.48, *p* = 0.02) than M-type. E-type significantly more breakfast skippers (21.8% vs. 10.1%, *p* = 0.02) than M-type. E-type was 1.7 times more likely to skip breakfast than M-type (CI 95%: 1.1–2.9, *p* = 0.02). Among breakfast skippers, the chronotype scores were negatively associated with dinner time (*β* = −0.17, *p* = 0.04).	-	No significant difference in BMI, waist circumference and abdominal fat between the chronotypes.
[42]	3 days food records Nutritional habits questionnaire	All E-type participants skipped a meal, while 11% of M-type and 14% of I-type skipped a meal. All chronotypes skipped breakfast the most; 60% E-type, 33% M-type and 44% I-type.	Among men, E-type compared to M-type significantly had: more calories (2450 kcal ± 625 vs. 1723 kcal ± 543, *p <* 0.001), carbohydrate (301.7 g ± 105.3 vs. 198.1 g ± 81.7, *p* = 0.02) and fats (99.3 g ± 26.7 vs. 69.7 g ± 18.9, *p <* 0.001) intake lower protein (12.5% ± 3.8 vs. 15.6% ± 2.8, *p* = 0.04) intake There was no significant difference between chronotype (men) in the consumption of fibre, cholesterol, vitamin A, E, B1, B2, B6 and C, sodium, potassium, calcium, magnesium, phosphorus, iron and zinc.	No significant difference in BMI between the chronotypes.
[43]	Semi-quantitative food frequency questionnaire	-	There was no association between chronotype scores with total energy and macronutrient intake (carbohydrate, protein and fat).	-
[46]	Night eating questionnaire (NEQ) Eating attitude test	E-type had significantly higher NEQ (night eating symptoms) and EAT score (higher score, higher severity of disordered eating) than other chronotypes (*p* < 0.001). Chronotype score (towards eveningness) was significantly negative associated with NEQ (*r* = −0.29, *p* < 0.01).	-	No significant association between BMI and chronotypes.
[47]	48 h diet recall	-	There was no significant difference between M (7709 kJ ± 97) and E-type (7679 kJ ± 215) in total daily energy intake. E-type compared to M-type significantly had: Less energy intake by 10:00 (16% vs. 20%, *p* < 0.001) More energy intake after 20:00 (18% vs. 11%, *p* < 0.001)	E-type (+1.4 kg ± 0.5) gained the most weight after seven years, but not significantly different from M- (+0.6 kg ± 0.2) and I-type (+0.8 kg ± 0.3).
[48]	Three 24 h diet recall	-	There was no association between chronotype and calorie intake.	-

ME, morningness–eveningness; MEQ, morningness–eveningness questionnaire Horne and Ostberg (1976); MCTQ, Munich chronotype questionnaire; CSM, composite scale of morningness; MSF, midpoint of sleep.

**Table 4:** The association of chronotype and specific food groups.

	Association of Chronotype and Food Group										
References	Grains	Legumes	Meat	Fish	Dairy Product	Fruits	Vegetables	Sweets	FAT & Oil	Caffeine	Alcohol
[23]	NA	NA	NA	NA	NA	NA	NA	NA	NA	+^a^	+ ^a^
[24]	NA	NA	NA	NA	NA	NA	NA	NA	NA	+ ^a,b^	+ ^a^
[26]	NA	NA	NA	NA	NA	NA	NA	NA	NA	NA	+ ^b^
[11]	− ^b^	− ^b^	+ ^b^	=	− ^b^	=	− ^b^	+ ^b^	+ ^b^	NA	+ ^b^
[27]	=	NA	=	=	=	=	− ^b^	=	NA	NA	NA
[13]	− ^b^	NA	=	− ^b^	=	=	− ^b^	+ ^b^	=	NA	+ ^b^
[32]	Pre										
	NA	NA	NA	NA	NA	NA	NA	NA	NA	=	=
	Post										
	NA	NA	NA	NA	NA	NA	NA	NA	NA	=	+ ^a^
[10]	Men										
	− ^b^	NA	=	− ^b^	=	=	=	NA	NA	NA	+ ^b^
	Women										
	− ^b^	NA	=	=	=	=	=	NA	NA	NA	+ ^b^
[33]	=	=	=	NA	=	=	+ ^b^	+ ^b^	− ^b^	NA	NA
[34]	=	NA	NA	NA	=	− ^a^	=	NA	+ ^a^	NA	+ ^a^
[8]	NA	NA	NA	NA	NA	− ^a^	− ^a^	NA	NA	NA	NA
[36]	=	NA	+ ^b^	NA	=	=	=	=	=	NA	NA
[37]	NA	NA	NA	NA	NA	NA	NA	NA	NA	+ ^a^	=
[43]	NA	NA	NA	NA	NA	− ^a^	− ^a^	NA	NA	+ ^a^	NA
[44]	NA	NA	NA	NA	NA	NA	NA	+ ^b^	NA	=	+ ^b^
[45]	NA	NA	NA	NA	NA	NA	NA	NA	NA	NA	=
[48]	+ ^b^	=	=	=	=	− ^b^	=	NA	=	NA	NA

^a^ significant difference between chronotype group; ^b^ significant association with chronotype score. + positive association with evening chronotype; − negative association with evening chronotype; = no difference/association

**Table 5:** Summary of findings.

	Morning Chronotype	Evening Chronotype
Dietary Behaviour		
Delay meal timing	−	+
Regular breakfast eater	+	−
Breakfast skipper	−	+
Excessive calorie during night	−	+
Food addiction	Limited	Limited
Feeling hunger	Limited	Limited
Longer eating duration	Limited	Limited
Watching TV during meal	Limited	Limited
Binge eating	Limited	Limited
Portion size	Limited	Limited
Skipped meal	Limited	Limited
Nutrient Intake		
Energy	=	=
Carbohydrate	=	=
Protein	+	−
Fat	=	=
Cholesterol	=	=
Fibre	=	=
Sucrose	-	+
Vitamins	Limited	Limited
Minerals	Limited	Limited
Food Group Intake		
Grains	Not enough evidence	Not enough evidence
Legumes	=	=
Meat	=	=
Fish	=	=
Dairy products	=	=
Fruits	Not enough evidence	Not enough evidence
Vegetables	+	−
Sweets	−	+
Fats & oil	Not enough evidence	Not enough evidence
Caffeine	−	+
Alcohol	−	+

+ positively related, − negatively related, = no differences.
